# Generalized Grey Target Decision Method for Mixed Attributes Based on Kullback-Leibler Distance

**DOI:** 10.3390/e20070523

**Published:** 2018-07-12

**Authors:** Jinshan Ma

**Affiliations:** School of Energy Science and Engineering, Henan Polytechnic University, Jiaozuo 454000, China; majshan@hpu.edu.cn

**Keywords:** Kullback-Leibler distance, mixed attributes, generalized grey target decision method, binary connection number, TOPSIS

## Abstract

A novel generalized grey target decision method for mixed attributes based on Kullback-Leibler (K-L) distance is proposed. The proposed approach involves the following steps: first, all indices are converted into index binary connection number vectors; second, the two-tuple (determinacy, uncertainty) numbers originated from index binary connection number vectors are obtained; third, the positive and negative target centers of two-tuple (determinacy, uncertainty) numbers are calculated; then the K-L distances of all alternatives to their positive and negative target centers are integrated by the Technique for Order Preference by Similarity to an Ideal Solution (TOPSIS) method; the final decision is based on the integrated value on a bigger the better basis. A case study exemplifies the proposed approach.

## 1. Introduction

The grey target decision method has been studied by many scholars since it was proposed by Deng [1]. Following the further research on decision-making, the indices of alternatives are extended from pure real values to mixed attribute values. Thus, this mixed attribute based grey target decision method is proposed to make it more applicable. The core of the grey target decision method is to obtain the target center distances and the alternatives to their target center, as the basis for decision-making. The certain number-based grey target decision method calculates the target center distance by distance method such as Euclidean distance and Mahalanobis distance [2,3]. The reported mixed attribute grey target decision method deals with target center distance in two ways: one is by distance including mainly Euclidean distance and other similar distances [4,5,6,7,8,9]; the other method is by vector-based distance, such as the generalized grey target decision method [10,11]. The generalized grey target decision method is different from the conventional one in that, during the calculation process, it obeys the principle of the conventional grey target decision method [10,11,12,13]. The tool for measuring the uncertainty of fuzzy numbers in mixed attribute based grey target decision method is needed to make decision-making more valuable in terms of its theoretical significance and practical application. Entropy is often used to measure uncertainty; thus, it is sure to be applied to the generalized grey target decision method involving fuzzy numbers. However, the Kullback-Leibler distance (K-L distance), originated from cross-entropy, has the ability to reflect the similarity of two discrete random distributions [14]. Now, cross-entropy has been widely used in many fields: Ioannis and George applied it to intuitionistic fuzzy information pattern recognition [15]. Li and Wu studied the alternative preference problem based on intuitionistic fuzzy cross-entropy [16]. Xia and Xu carried out group decision-making which comprises intuitionistic fuzzy information [17]. Smieja and Geiger studied the cluster problem confined by information using cross-entropy [18]. Tang et al. proposed an optimization algorithm based on cross-entropy [19].

The principle of this proposed approach goes as follows: all indices of alternatives are first converted into binary connection number vectors and also divided into those of deterministic terms and uncertain terms based on the previous method. Then the deterministic terms and uncertain terms of positive target centers and negative target centers under each attribute can be obtained. Next, the two-tuple (determinacy, uncertainty) numbers originated from index binary connection number vectors are deduced. Following that, the K-L distances of all alternatives to their positive and negative target centers are integrated by using the TOPSIS method: the final decision is based on the integrated value for which the bigger is the better.

## 2. Basic Theory

### 2.1. Fuzzy Number

**Definition** **1.** Let **R** be a real domain; if $\tilde{x}$ denotes a fuzzy number, [x^L^, x^U^], [x^L^, x^M^, x^U^] and [x^L^, x^M^, x^N^, x^U^] are the expressions of $\tilde{x}$ called the interval number, triangular fuzzy number and trapezoidal fuzzy number, respectively, where x^L^, x^M^, x^N^ and x^U^ satisfy 0 < x^L^ < x^M^ < x^N^ < x^U^ ∈ **R** [20,21].

### 2.2. Binary Connection Number

**Definition** **2.** Let **R** be a real domain; A + Bi is called a binary connection number, where A represents the deterministic term, B is the uncertain term and i is a variable term unifying the determinacy and uncertainty of a fuzzy number and A, B ∈ R, i ∈ [−1, 1].

**Definition** **3.** $\bar{x}$ *and* v* are the mean value and deviation value of the n* (*n ≥* 2) *parameters of* $\tilde{x}$ *respectively, then:*

(1) $$u(\bar{x},v)=A+Bi=\bar{x}+vi(i\in [-1,1])$$

*is called a mean value-deviation value connection number. Where* $\bar{x}$*,* S*,* ms*, and* v are calculated by use of Equations (2)–(5):

(2) $$\bar{x}=\frac{1}{n}\sum_{j=1}^{n}x_{j}$$

(3) $$S=\sqrt{\frac{1}{\left(n-1\right)}\sum_{j=1}^{n}(x_{j}-\bar{x}{)}^{2}}$$

(4) $$ms=\max\left\{\left|x^{L}-\bar{x}\right|,\left|x^{U}-\bar{x}\right|\right\}$$

(5) v=min{S,ms}

*where* $x_{j}(j=1,\cdots ,n)$ *is the jth parameter of the fuzzy number* $\tilde{x}$*,* $\bar{x}$ *is the mean value of the parameters,* S *denotes the standard deviation of the parameters, ms is the maximum deviation of the parameters,* v *is the minimum of* S *and* ms*, x^L^ and x^U^ are the fuzzy number’s lower limits and upper limits, respectively [10,22].*

**Definition** **4.** *The mutual interaction of the mean value* $\bar{x}$ *and the deviation value* v *(standard deviation or maximum deviation) of the binary connection number* $u(\bar{x},v)$ *can be mapped to the determinacy-uncertainty space (**D**-**U** space). If* $u(\bar{x},v)=\bar{x}+vi$ *represents the vector in **D**-**U** space, then i only denotes the signal of the uncertain term without representing the changeable value [20,21].*

**Figure 1:**
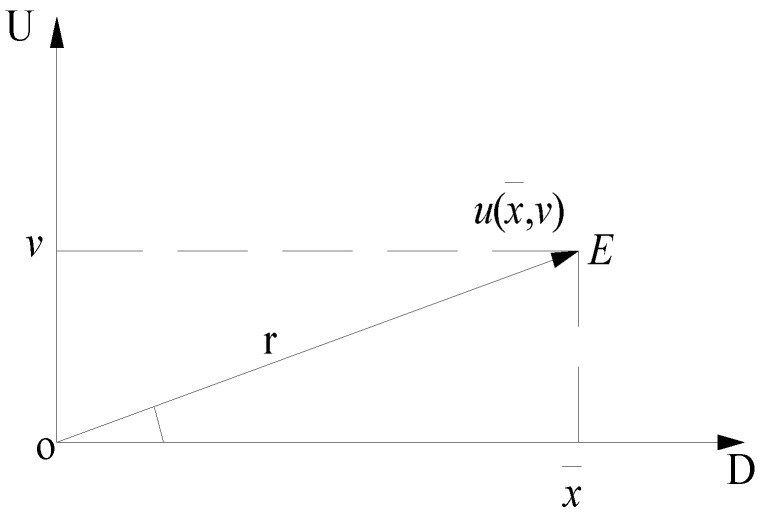
Determinacy-uncertainty space [22].

Figure 1 shows a ***D****-**U*** space. The ***U****-*axis represents the relative uncertainty measure, while the ***D****-*axis denotes the relative deterministic measure. As seen from Figure 1, $\bar{x}$ and S interact with each other and the space reflection is the vector $\bar{OE}$ from ***O*** to ***E*** and the degree of interaction represents the modulus of vector $\bar{OE}$, denoted by *r*.

### 2.3. Kullback-Leibler Distance

**Definition** **5.** *Kullback-Leibler distance [14,15]. Let $X={\left(x_{1},x_{2},\cdots ,x_{m}\right)}^{Τ}$ and $Y={\left(y_{1},y_{2},\cdots ,y_{m}\right)}^{Τ}$ be two vectors, where $x_{j},y_{j}\geq 0,j=1,2,\ldots ,m$, $1=\sum_{j=1}^{m}x_{j}\geq y_{j}$, then the K-L distance of X and Y is given by Equation (6).*

(6) $$H(X,Y)=\sum_{j=1}^{m}x_{j}\ln\frac{x_{j}}{y_{j}}$$

H(X,Y) *exhibits the following characteristics:*

*(1)*  $H(X,Y)=\sum_{j=1}^{m}x_{j}\ln\frac{x_{j}}{y_{j}}\geq 0$;
*(2)*  $H(X,Y)=\sum_{j=1}^{m}x_{j}\ln\frac{x_{j}}{y_{j}}=0$, *when and only, when* $x_{j}=y_{j},\forall j$.

*If $x_{j}\neq 0,y_{j}=0$ then H(X,Y)→∞. So, the original K-L distance needs to be improved. The revised version of the K-L distance is as follows:*

(7) $$K(X,Y)=H(X,\frac{X+Y}{2})=\sum_{j=1}^{n}x_{j}\ln\frac{x_{j}}{\frac{1}{2}(x_{j}+y_{j})}$$

**Definition** **6.** *Comprehensive weighted K-L distance. Let the symbols* $S={\left(\left(x_{1},y_{1}),(x_{2},y_{2}),\ldots ,(x_{m},y_{m}\right)\right)}^{Τ}$ *and* $E={\left(\left(p_{1},q_{1}),(p_{2},q_{2}),\ldots ,(p_{m},q_{m}\right)\right)}^{Τ}$*, refer to the vectors of two-tuple (determinacy, uncertainty) numbers, where* $x_{j},y_{j},p_{j},q_{j}\geq 0,j=1,2,\ldots ,m$*, are two-tuple (determinacy, uncertainty) numbers under the same attributes in S and E respectively. Denote the weight vector *W *by* $W={\left(w_{1},w_{2},\cdots ,w_{m}\right)}^{Τ}$*,* $w_{j}>0$*,* j=1,2,…,m *and assume that for the two-tuples* S *and* E *the following normalization condition is satisfied:*

(8) $$\sum_{j=1}^{m}w_{j}(x_{j}+y_{j})\geq \sum_{j=1}^{m}w_{j}(p_{j}+q_{j})$$

The comprehensive weighted K-L distance $H_{W}(S,E)$ can be calculated using the following equation:

(9) $$H_{W}(S,E)=\sum_{j=1}^{m}w_{j}\left(x_{j}\ln\frac{x_{j}}{p_{j}}+y_{j}\ln\frac{y_{j}}{q_{j}}\right)$$

Then the function $H_{W}(S,E)$ has the following properties:

(1) $H_{W}(S,E)\geq 0$;
(2) $H_{W}(S,E)=0$, when and only, when S=E, or what amounts to the same, $x_{j}=p_{j}$ and $y_{j}=q_{j}$,j=1,2,…,m;
(3) when $x_{j}=p_{j}=0$ or $y_{j}=q_{j}=0$, then, by definition, $x_{j}\ln\frac{x_{j}}{p_{j}}=y_{j}\ln\frac{y_{j}}{q_{j}}=0$.

The assertions in (1) and (2) can be proved as follows. We assume that $p_{j}>0$ and $q_{j}>0$ for j=1,2,…,m. In the following sequence of (in-) equalities we apply the convexity of the function u↦ulnu,u>0, or what amounts to the same the log-sum inequality, also called Gibb’s inequality:

$$\begin{array}{ll} H_{W}(S,E) & =\sum_{j=1}^{m}w_{j}\left(x_{j}\ln\frac{x_{j}}{p_{j}}+y_{j}\ln\frac{y_{j}}{q_{j}}\right)\\ & =\left(\sum_{k=1}^{m}w_{k}p_{k}\right)\sum_{j=1}^{m}\frac{w_{j}p_{j}}{\sum_{k=1}^{m}w_{k}p_{k}}\frac{x_{j}}{p_{j}}\ln\frac{x_{j}}{p_{j}}\\ & +\left(\sum_{k=1}^{m}w_{k}q_{k}\right)\sum_{j=1}^{m}\frac{w_{j}q_{j}}{\sum_{k=1}^{m}w_{k}q_{k}}\frac{y_{j}}{q_{j}}\ln\frac{y_{j}}{q_{j}} \end{array}$$

(the function u↦ulnu,u>0, is convex)

$$\begin{array}{l} \geq \left(\sum_{k=1}^{m}w_{k}p_{k}\right)\sum_{j=1}^{m}\frac{w_{j}p_{j}}{\sum_{k=1}^{m}w_{k}p_{k}}\frac{x_{j}}{p_{j}}\ln\frac{\sum_{j=1}^{m}w_{j}x_{j}}{\sum_{k=1}^{m}w_{k}p_{k}}\\ +\left(\sum_{k=1}^{m}w_{k}q_{k}\right)\sum_{j=1}^{m}\frac{w_{j}q_{j}}{\sum_{k=1}^{m}w_{k}q_{k}}\frac{y_{j}}{q_{j}}\ln\frac{\sum_{j=1}^{m}w_{j}y_{j}}{\sum_{k=1}^{m}w_{k}q_{k}}\\ =\left(\sum_{j=1}^{m}w_{j}x_{j}\right)\ln\frac{\sum_{j=1}^{m}w_{j}x_{j}}{\sum_{k=1}^{m}w_{k}p_{k}}+\left(\sum_{j=1}^{m}w_{j}y_{j}\right)\ln\frac{\sum_{j=1}^{m}w_{j}y_{j}}{\sum_{k=1}^{m}w_{k}q_{k}}. \end{array}$$

Put $x=\sum_{j=1}^{m}w_{j}x_{j}$, $y=\sum_{j=1}^{m}w_{j}y_{j}$, $p=\sum_{k=1}^{m}w_{k}p_{k}$ and $q=\sum_{k=1}^{m}w_{k}q_{k}$.

Then the inequality in property (1) implies

$$\begin{array}{ll} H_{W}(S,E) & \geq x\ln\frac{x}{p}+y\ln\frac{y}{q}\\ & =\left(p+q\right)\left\{\frac{p}{p+q}\frac{x}{p}\ln\frac{x}{p}+\frac{q}{p+q}\frac{y}{q}\ln\frac{y}{q}\right\} \end{array}$$

(apply once more the convexity of the function u↦ulnu,u>0)

$$\geq \left(p+q\right)\frac{x+y}{p+q}\ln\frac{x+y}{p+q}=\left(x+y\right)\ln\frac{x+y}{p+q}.$$

The following inequality is true for u>0:lnu≤u−1. Hence, we infer:

$$\begin{array}{ll} \left(x+y\right)\ln\frac{x+y}{p+q} & =-\left(x+y\right)\ln\frac{p+q}{x+y}\\ & \geq -\left(x+y\right)\left\{\frac{p+q}{x+y}-1\right\}\\ & =x+y-\left(p+q\right)\geq 0. \end{array}$$

The final inequality follows from the normalization conditions on S and E. This shows the inequality in property (1). If $H_{W}(S<E)=0$, then all the previous inequalities are in fact equalities. This can only be true provided $x_{j}=p_{j}$ and $y_{j}=q_{j}$ for j=1,2,…,m. This is kind of a converse to the Jensen inequality, or as it is presented here the log-sum inequality or Gibb’s inequality. Observe that the proofs of properties (1) and (2) can also be adapted to the situation where some of $p_{j}$’s or some of the $q_{j}$’s are zero. Essentially speaking the same proof works by summing over those 1≤j≤m for which $p_{j}\neq 0$ or for which $q_{j}\neq 0$.

However, if the condition for S and E in Equation (8) is not satisfied, then $H_{W}(S,E)<0$ may occur, thus an improved version of which is given as follows.

(10) $$K_{W}(S,E)=\sum_{j=1}^{m}w_{j}\left(x_{j}\left|\ln\frac{x_{j}}{p_{j}}\right|+y_{j}\left|\ln\frac{y_{j}}{q_{j}}\right|\right)$$

In Equation (10), $K_{W}(S,E)$ has the same characteristics as $H_{W}(S,E)$ but it can solve the special problem that the condition in Equation (8) is not satisfied.

## 3. Generalized Grey Target Decision Method for Mixed Attributes Based on the K-L Distance

Let $C=\left\{C_{1},C_{2},\cdots ,C_{n}\right\}$, $A=\left\{A_{1},A_{2},\cdots ,A_{m}\right\}$ and $W={\left(w_{1},w_{2},\cdots ,w_{m}\right)}^{Τ}$ be an alternative set, attribute set and weight vector of index attributes respectively, then the index of alternative $C_{s}$ under attribute $A_{t}$ is $v_{st}(s=1,2,\cdots ,n;\;t=1,2,\cdots ,m)$.

### 3.1. Transformation of Index Values into Binary Connection Numbers

Different types of index values can be converted into binary *A* + *Bi* connection numbers regarded as vectors in ***D***-***U*** space using Equations (1)–(5). It is noteworthy that the converted binary connection number for real number is of the form *A* + 0*i*, which means that the deterministic term is the real number itself and the uncertain term is 0*i*. The transformed index vector can be expressed as $U_{st}=A_{st}+B_{st}i(s=1,2,\cdots ,n;\;t=1,2,\cdots ,m)$.

### 3.2. Determination of the Target Centre Index Vectors

Having achieved the binary connection numbers converted from all index values, $U_{st}=A_{st}+B_{st}i(s=1,2,\cdots ,n;\;t=1,2,\cdots ,m)$, which can also be denoted by the two-tuple number $U_{st}=(A_{st},B_{st}),(s=1,2,\cdots ,n;\;t=1,2,\cdots ,m)$. The benefit type index set and cost type index set, are denoted by *J*^+^ and *J*^−^, respectively. Then the positive and negative target center index vectors of two-tuple (determinacy, uncertainty) denoted by $C_{t}^{+}$ and $C_{t}^{-}$ can be obtained using Equations (11) and (12).

The positive target center index of two-tuple (determinacy, uncertainty) is as follows:

(11) $$C_{t}^{+}=\left\{ \begin{array}{l} \left(\max\left\{A_{st}\right\},\min\left\{B_{st}\right\}\right),U_{st}\in J^{+}\}\\ \left(\min\left\{A_{st}\right\},\min\left\{B_{st}\right\}\right),U_{st}\in J^{-}\} \end{array}\right.,s=1,2,\cdots ,n,\;t=1,2,\cdots ,m$$

The negative target center index of two-tuple (determinacy, uncertainty) is as follows:

(12) $$C_{t}^{-}=\left\{ \begin{array}{l} \left(\min\left\{A_{st}\right\},\max\left\{B_{st}\right\}\right),U_{st}\in J^{+}\}\\ \left(\max\left\{A_{st}\right\},\max\left\{B_{st}\right\}\right),U_{st}\in J^{-}\} \end{array}\right.,s=1,2,\cdots ,n,\;t=1,2,\cdots ,m$$

Equation (11) indicates that the positive target center index of two-tuple (determinacy, uncertainty) number under attribute $A_{t}$ is such that the index vector corresponding to the maximum term and minimum term for benefit-type indices and that of the minimum term and minimum term is used for cost-type indices. Equation (12) represents the fact that the negative target center index the two-tuple (determinacy, uncertainty) number under attribute $A_{t}$ is such that the index vector corresponding to the minimum term and maximum term is used for benefit-type indices and that of the maximum term and maximum term is used for cost-type indices.

### 3.3. Normalization of All Alternative Indices

The index vectors of all alternatives $U_{st}=A_{st}+B_{st}i(s=1,2,\cdots ,n;\;t=1,2,\cdots ,m)$ and target center index vectors $U_{ct}=A_{ct}+B_{ct}i(c=n+1;\;t=1,2,\cdots ,m)$ can be expressed as vectors of two-tuple (deterministic degree, uncertainty degree) numbers:

(13) $$a_{st}=\frac{A_{st}}{A_{st}+B_{st}},b_{st}=\frac{B_{st}}{A_{st}+B_{st}},(s=1,2,\cdots ,n+1;\;t=1,2,\cdots ,m)$$

In Equation (13), $a_{st}$ and $b_{st}$ denote respectively the deterministic degree and uncertainty degree under the same attribute in normalized binary connection numbers. Then the vector of two-tuple (deterministic degree, uncertainty degree) number can be given as ${\left(\left(a_{s1},b_{s1}),\;(a_{s2},b_{s2}),\;\ldots ,\;(a_{sm},\;b_{sm}\right)\right)}^{Τ}$. It should be noted that a real number cannot be normalized in this step, or an error will occur when computing the uncertain term of real numbers as they are all zero under the same attribute.

The $a_{st}$ and $b_{st}$ in a two-tuple (deterministic degree, uncertainty degree) number $\left(a_{st},b_{st}\right)$ should be normalized further for they are incomparable under different attributes. The normalization equation is as follows:

(14) $$a_{st}^{\prime }=\frac{a_{st}}{\sum_{s=1}^{n}a_{st}},b_{st}^{\prime }=\frac{b_{st}}{\sum_{s=1}^{n}b_{st}},s=1\ldots n;\;t=1\ldots m$$

In Equation (14), $a_{st}^{\prime }$ and $b_{st}^{\prime }$ are the normalized deterministic term and uncertainty term respectively in the two-tuple number.

### 3.4. Integration by TOPSIS Method

The closeness of comprehensive weighted K-L distance is used to judge the alternatives with the full consideration of the effects on each alternative on its positive and negative target centers. The TOPSIS method has been used extensively since it was proposed [23]. Let $r_{i}^{P}$ and $r_{i}^{N}$ represent, respectively, the positive comprehensive weighted K-L distance and negative comprehensive weighted K-L distance, then the closeness of the comprehensive weighted K-L distance can be obtained by using Equation (15):

(15) $$C_{i}=\frac{r_{i}^{N}}{r_{i}^{P}+r_{i}^{N}},i=1\ldots n.$$

The decision-making could be based on $C_{i}$ for which, the larger the better.

### 3.5. Decision-Making Steps

**Figure 2:**
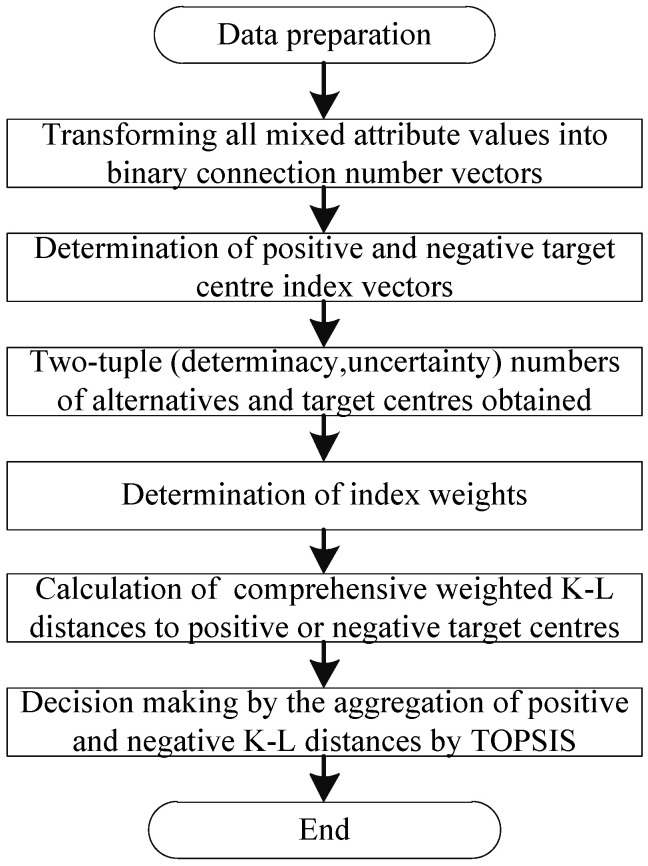
The K-L distance-based generalized grey target decision method.

The procedure of generalized grey target decision method based on K-L distance is shown in Figure 2; the detailed steps therein are as follows:

(1) All indices of alternatives are converted into binary connection number vectors and comprised of two-tuple (determinacy, uncertainty) numbers by using Equations (1)–(5).
(2) The positive and negative target center indices of two-tuple (determinacy, uncertainty) number under all attributes are determined by using Equations (11) and (12).
(3) All two-tuple (determinacy, uncertainty) numbers are transformed into two-tuple (deterministic degree, uncertainty degree) numbers by using Equation (13) and they can also be normalized using the linear method given in Equation (14).
(4) The weights of all index attributes are calculated.
(5) The comprehensive weighted K-L distances of normalized two-tuple (deterministic degree, uncertainty degree) numbers between all alternatives and the target center are calculated by using Equation (9) or Equation (10); then the closeness of all alternatives can be obtained by use of the TOPSIS method and Equation (15).
(6) The decision-making is realized according to the closeness of each alternative for which, the larger the better.

## 4. Case Study

### 4.1. Data Resource

To evaluate tactical missiles, six indices including hit accuracy (km), warhead payload (kg), mobility (km·h^−1^), price (10^6^ g), reliability and maintainability are denoted by *A*_1_ to *A*_6_ [8]. For all data types of attributes, *A*_1_ and *A*_2_ are real numbers, *A*_3_ and *A*_4_ are interval numbers and *A*_5_ and *A*_6_ are triangular fuzzy numbers. Among these attributes *A*_1_ and *A*_4_ are cost type indices and the others are benefit type indices. There are four feasible alternatives denoted by *C*_1_ to *C*_4_. The data are summarized in Table 1.

### 4.2. Decision-Making Process

#### 4.2.1. Calculation of the Parameters of Binary Connection Number of All Alternatives

The parameters of binary connection number of all alternatives can be calculated from the data in Table 1 by using Equations (1)–(5): the results are shown in Table 2.

#### 4.2.2. Translate All Index Values into Binary Connection Number Vectors

All index values can be transformed into index vectors using Equations (1)–(5) based on the data listed in Table 2. Table 3 lists the binary connection numbers as converted from all indices.

Then the two-tuple (determinacy, uncertainty) numbers shown in Table 4 are converted from index binary connection number vectors as shown in Table 3.

#### 4.2.3. Determination of the Two-Tuple Numbers of Positive and Negative Target Centers

The vectors of two-tuple numbers of the positive target center are calculated as ((1.8, 0), (540, 0), (55.5, 0.5), (4.7, 0.5), (0.7, 0.1), (0.9, 0.1)) by using Equation (11).

The vectors of two-tuple numbers of the negative target center are obtained as ((2.5, 0), (480, 0), (35, 5), (5.5, 0.5), (0.3, 0.1), (0.5, 0.1)) by using Equation (12).

#### 4.2.4. Normalization of the Two-Tuple (Deterministic Degree, Uncertainty Degree) Numbers

Two-tuple (deterministic degree, uncertainty degree) numbers of alternative indices and target center indices can be normalized by using Equation (14), the results are as summarized in Table 5.

In Table 5, $a_{st}$ and $b_{st}$ in $\left(a_{st},\;b_{st}\right)$ represent, respectively, the deterministic term and uncertain term of the same index. If an index is a real number, then $b_{st}$ is zero, for example, the indices under attribute *A*_1_ and *A*_2_ are all real numbers. The symbols *NC*_P_ and *NC*_N_ denote the normalized two-tuple (deterministic degree, uncertainty degree) number of the positive target center and that of a negative target center respectively.

#### 4.2.5. Determination of the Closeness of Comprehensive Weighted K-L Distances of Positive and Negative Centers

Given that the weight vector *W* = (0.1818, 0.2017, 0.1004, 0.2124, 0.1618, 0.1419), the comprehensive weighted K-L distances of all alternatives to their positive target center indices are *KL^+^* = (0.0265, 0.1910, 0.0804, 0.1402) from Equation (10). The comprehensive weighted K-L distances of all alternatives to their negative target center indices are calculated as *KL^−^* = (0.0607, 0.0152, 0.0615, 0.0394) by use of Equation (10). Here, Equation (10) is used to calculated the *KL^+^* and *KL^−^*, because the condition in Equation (8) is not satisfied provided the given data for *KL^−^*. Then the closeness of positive K-L distance and negative K-L distance is calculated as *TS* = (0.6961, 0.0737, 0.4334, 0.2194) by the TOPSIS method using Equation (15). The final decision-making can be made in accordance with the closeness with the larger value being the better alternative as follows: $S_{1}>S_{3}>S_{4}>S_{2}$.

### 4.3. Analysis and Discussion

For comparison, *W* = (0.1818, 0.2017, 0.1004, 0.2124, 0.1618, 0.1419) is given; using the approach in [10] the comprehensive weighted proximity (CWP) is: *I_CWP_* = (0.2023, 0.2928, 0.2354, 0.2695). According to the rule stating that the smaller the proximity the better the alternative, the ranking of the alternatives is as follows: $S_{1}>S_{3}>S_{4}>S_{2}$*.* Table 6 summarizes the comparison of the proximity based method and the proposed method.

Table 6 lists the results calculated by two kinds of approaches: the K-L distance-based method and a vector-based method. The K-L distance-based method offers two ways in which to fulfil the decision-making task: one is to obtain the comprehensive weighted K-L distance based on the positive target center. The decision is made on the basis of the smaller the value the better. The other depends on the closeness of the two kinds of comprehensive weighted K-L distances such that all alternatives to their positive and negative target centers are covered. The ranking of the alternatives is on a larger-the-better basis. The vector-based method, which makes decision mainly by comprehensive weighted proximity, depends on a value for which the smaller the better. Through comparison, decision-making by the proposed method is in accordance with that by the vector-based method; however, the proposed method has one difference with the vector-based method in the principle governing its decision-making. The similarity of, and difference between, the two methods are analyzed next.

The two methods have a similarity: the proposed method and the method reported in [10] all transform different types of data into a binary connection number which can be handled in the same way. In brief, the binary connection number is the main tool used when dealing with mixed attribute values. The difference between the two methods is: the proposed method adopts the comprehensive weighted K-L distance to determine the ranking of alternatives, which makes the decision from the prospect of entropy as a measure of the uncertainty; while the method in [10] uses the comprehensive weighted proximity to determine this ranking, as it works from the viewpoint of the similarity of the vectors.

## 5. Conclusions

With this research, we arrive at the following conclusions:

(a) A novel generalized grey target decision method is presented and this method uses the binary connection number and K-L distance as its bases.
(b) The decision making is based on the comprehensive weighted K-L distance.
(c) The calculation result is in agreement with the reported method; however, the proposed method makes its decision based on K-L distance, as this can measure the uncertainty therein. Thus, the proposed method is valuable with regard to its theoretical significance and benefits conferred in practical application.

## Figures and Tables

**Table 1:** Index values of every alternative.

*S*_i_	*A*_1_	*A*_2_	*A*_3_	*A*_4_	*A*_5_	*A*_6_
***S*_1_**	2.0	500	[55, 56]	[4.7, 5.7]	[0.4, 0.5, 0.6]	[0.8, 0.9, 1.0]
***S*_2_**	2.5	540	[30, 40]	[4.2, 5.2]	[0.2, 0.3, 0.4]	[0.4, 0.5, 0.6]
***S*_3_**	1.8	480	[50, 60]	[5, 6]	[0.6, 0.7, 0.8]	[0.6, 0.7, 0.8]
***S*_4_**	2.2	520	[35, 45]	[4.5, 5.5]	[0.4, 0.5, 0.6]	[0.4, 0.5, 0.6]

**Table 2:** Average values, standard deviations and maximum deviations of all indices.

*S*_i_	*A*_1_	*A*_2_	*A*_3_	*A*_4_	*A*_5_	*A*_6_
***S*_1_**	2.0/0/0	500/0/0	55.5/0.7071/0.5	5.2/0.7071/0.5	0.5/0.1/0.1	0.9/0.1/0.1
***S*_2_**	2.5/0/0	540/0/0	35/7.0711/5	4.7/0.7071/0.5	0.3/0.1/0.1	0.5/0.1/0.1
***S*_3_**	1.8/0/0	480/0/0	55/7.0711/5	5.5/0.7071/0.5	0.7/0.1/0.1	0.7/0.1/0.1
***S*_4_**	2.2/0/0	520/0/0	40/7.0711/5	5/0.7071/0.5	0.5/0.1/0.1	0.5/0.1/0.1

**Note:** “a/b/c” in Table 2 denotes “average value/standard deviation/maximum deviation”.

**Table 3:** Index binary connection number vectors transformed from index values.

*S*_i_	*A*_1_	*A*_2_	*A*_3_	*A*_4_	*A*_5_	*A*_6_
***S*_1_**	2.0 + 0*i*	500 + 0*i*	55.5 + 0.5*i*	5.2 + 0.5*i*	0.5 + 0.1*i*	0.9 + 0.1*i*
***S*_2_**	2.5 + 0*i*	540 + 0*i*	35 + 5*i*	4.7 + 0.5*i*	0.3 + 0.1*i*	0.5 + 0.1*i*
***S*_3_**	1.8 + 0*i*	480 + 0*i*	55 + 5*i*	5.5 + 0.5*i*	0.7 + 0.1*i*	0.7 + 0.1*i*
***S*_4_**	2.2 + 0*i*	520 + 0*i*	40 + 5*i*	5 + 0.5*i*	0.5 + 0.1*i*	0.5 + 0.1*i*

**Table 4:** Two-tuple numbers transformed from index binary connection number vectors.

*S*_i_	*A*_1_	*A*_2_	*A*_3_	*A*_4_	*A*_5_	*A*_6_
***S*_1_**	(2.0, 0)	(500, 0)	(55.5, 0.5)	(5.2, 0.5)	(0.5, 0.1)	(0.9, 0.1)
***S*_2_**	(2.5, 0)	(540, 0)	(35, 5)	(4.7, 0.5)	(0.3, 0.1)	(0.5, 0.1)
***S*_3_**	(1.8, 0)	(480, 0)	(55, 5)	(5.5, 0.5)	(0.7, 0.1)	(0.7, 0.1)
***S*_4_**	(2.2, 0)	(520, 0)	(40, 5)	(5, 0.5)	(0.5, 0.1)	(0.5, 0.1)

**Table 5:** Normalized two-tuple numbers of all alternatives and target center indices.

*NS*_i_	*A*_1_	*A*_2_	*A*_3_	*A*_4_	*A*_5_	*A*_6_
***NS*_1_**	(0.2353, 0)	(0.2451, 0)	(0.2699, 0.0272)	(0.2505, 0.2449)	(0.2532, 0.2353)	(0.2615, 0.1791)
***NS*_2_**	(0.2941, 0)	(0.2647, 0)	(0.2383, 0.3807)	(0.2482, 0.2685)	(0.2278, 0.3529)	(0.2421, 0.2985)
***NS*_3_**	(0.2118, 0)	(0.2353, 0)	(0.2497, 0.2538)	(0.2517, 0.2327)	(0.2658, 0.1765)	(0.2542, 0.2239)
***NS*_4_**	(0.2588, 0)	(0.2549, 0)	(0.2421, 0.3384)	(0.2496, 0.2539)	(0.2532, 0.2353)	(0.2421, 0.2985)
***NC*_P_**	(0.2118, 0)	(0.2647, 0)	(0.2699, 0.0272)	(0.2482, 0.2685)	(0.2658, 0.1765)	(0.2615, 0.1791)
***NC*_N_**	(0.2941, 0)	(0.2353, 0)	(0.2383, 0.3807)	(0.2517, 0.2327)	(0.2278, 0.3529)	(0.2421, 0.2985)

**Table 6:** Comparison of the results of two decision methods.

	Comprehensive Weighted K-L Distance Method				Comprehensive Weighted Proximity Method	
	*KL^+^*	Rank	*TS*	Rank	*I_CWP_*	Rank
*S*_1_	0.0265	1	0.6961	1	0.2023	1
*S*_2_	0.1910	4	0.0737	4	0.2928	4
*S*_3_	0.0804	2	0.4334	2	0.2354	2
*S*_4_	0.1402	3	0.2194	3	0.2695	3
